# Baculovirus-Assisted Production of *Bartonella bacilliformis* Proteins: A Potential Strategy for Improving Serological Diagnosis of Carrion’s Disease

**DOI:** 10.3390/pathogens13080690

**Published:** 2024-08-15

**Authors:** Lizbeth Sally Vilca-Machaca, Karen Daphne Calvay-Sanchez, Yanina Zarate-Sulca, Victor Jimenez-Vasquez, Pablo Ramirez, Giovanna Mendoza-Mujica

**Affiliations:** 1 Faculty of Biological Sciences, Universidad Nacional Mayor de San Marcos, Lima 15081, Peru; 2 Laboratory of Vector-Borne and Zoonotic Bacterial Diseases, National Institute of Health, Lima 15072, Peru

**Keywords:** *Bartonella bacilliformis*, Prot_689, Prot_504, BEVS system, flash BAC ULTRA technology, recombinant proteins

## Abstract

Carrion’s disease, caused by *Bartonella bacilliformis*, is a neglected tropical disease prevalent in the Andean region of South America. Without antimicrobial treatment, this disease has a mortality rate of up to 88% in infected patients. The most common method for diagnosing *B. bacilliformis* infection is serological testing. However, the current serological assays are limited in sensitivity and specificity, underscoring the need for the development of novel and more accurate diagnostic tools. Recombinant proteins have emerged as promising candidates to improve the serological diagnosis of Carrion’s disease. So, we focused on evaluating the conditions for producing two previously predicted proteins of *B. bacilliformis* using the baculovirus–insect cell expression system, mainly the flashBAC ULTRA technology. We assessed various parameters to identify the conditions that yield the highest protein production, including cell lines, temperature, and hours post-infection (hpi). The results showed that the expression conditions for achieving the highest yields of the Prot_689 and Prot_504 proteins were obtained using High Five™ cells at 21 °C and harvesting at 120 hpi. Subsequently, the seroreactivity of recombinant proteins was evaluated using positive sera from patients diagnosed with Carrion’s disease. These findings offer valuable insights into the production conditions of *B. bacilliformis* recombinant proteins using the baculovirus system, which could significantly contribute to developing more precise diagnostic tools for Carrion’s disease. Therefore, this research provides implications for improving diagnostics and potentially developing therapeutic strategies.

## 1. Introduction

*Bartonella bacilliformis* is a gram-negative bacterium and the causative agent of Carrion’s disease, a neglected tropical disease prevalent in small Andean communities in Peru and other countries like Colombia, Ecuador, Bolivia, and Chile [1,2,3,4]. Carrion’s disease has two clinical phases with a significant impact on public health in low-resource populations from endemic regions [1,2]. The acute phase, known as “Oroya fever”, is characterized by severe hemolytic anemia and can be fatal, with an untreated mortality rate of up to 88% [2,5,6,7]. The chronic phase, called “Peruvian wart”, occurs after the acute infection and is non-fatal but leads to debilitating vascular cutaneous lesions and significantly diminishes the patients’ well-being [7].

Peru is the country most affected by Carrion’s disease and the only one with reported cases in the last decade [2,8,9]. The department of Ancash has the highest endemicity, with the seroprevalence among the general population of these areas being greater than 60% [2,8], as evaluated using the slide agglutination test [8].

The detection of *Bartonella bacilliformis* is carried out through blood smear, bacteriological culture, and PCR, each with its own set of limitations such as low sensitivity, being time-consuming, difficulty for routine practice, and false negatives [10,11]. Serological methods offer an alternative approach for the indirect diagnosis of Carrion’s disease by detecting antibodies against *B. bacilliformis* [12], thus overcoming the limitations of traditional diagnostic methods. The Western blot technique, while highly sensitive and specific, is complex and costly [13,14], making it difficult to implement in regional laboratories. In contrast, the enzyme-linked immunosorbent assay (ELISA) provides a rapid serological diagnosis and is cost-effective and easy to implement [12,15].

The Laboratory of Vector-Borne and Zoonotic Bacterial Diseases (LRNMEZOB) developed an indirect ELISA for diagnosing Carrion’s disease utilizing soluble *B. bacilliformis* antigen prepared from bacterial lysates. Despite acceptable sensitivity (IgG 93.3% and IgM 90.0%) and low specificity (IgG 88% and IgM 84%) as reported in internal institutional documentation (MET-CNSP-183 and MET-CNSP-184, National Institute of Health, Lima, Peru), the ELISA could produce a cross-reactivity with other infections produced by other *Bartonella* species and unrelated pathogens [12].

The ELISA technique, which utilizes recombinant antigens for the diagnosis of infectious diseases, has been widely documented. These assays often demonstrate high sensitivity and specificity, surpassing the performance of immunoassays that deploy total antigens [16,17,18,19,20]. This is because recombinant antigens are defined entities with potentially fewer epitopes compared to total antigen preparations, which helps reduce cross-reactivity [19,20]. This characteristic has been reported as particularly beneficial in the diagnosis of diseases caused by pathogens such as *Brucella melitensis* [16], *Burkholderia pseudomallei* [17], and *Orientia tsutsugamushi* [18], where specificity and sensitivity are crucial for accurate detection and subsequent management of these infectious diseases.

Explorations into recombinant proteins from *B. bacilliformis* have been conducted, yet to date, none has been adopted for diagnostic applications [21,22,23]. These recombinant antigens were produced using heterologous systems, such as the *Escherichia coli* bacterial system. However, this system has drawbacks, including challenges in achieving high yields of recombinant proteins, post-translational modifications, the formation of inclusion bodies, inefficient protein translocation, or correct protein folding, in addition to difficulties in scaling production efficiently [24,25].

In contrast, the use of baculovirus expression systems, such as flashBAC ULTRA, offers significant advantages over the limitations associated with the *E. coli* system [26]. flashBAC ULTRA is based on homologous recombination of the transfer vector pBac1, which contains the gene of interest along with the flashBAC sequence for the generation of recombinant baculoviruses. flashBAC technology, which includes deletions in secretion genes, enhances the expression of extracellular proteins by removing unnecessary genes. This optimization allows for improved secretion and expression of complex proteins, including membrane and highly processed secreted proteins [27,28]. Its application has been extensively utilized to produce recombinant proteins in insect cells, including antigens for serological diagnosis of infectious diseases, vaccines, and gene therapy vectors [29,30,31]. This advanced system facilitates efficient expression in insect cells, such as *Spodoptera frugiperda* (Sf9) [32] and *Trichoplusia ni BTI-Tn-5B1-4* (High Five™) [33], overcoming the drawbacks associated with other systems and marking a significant advancement in recombinant protein expression biotechnology.

A previous bioinformatic analysis identified potential antigenic proteins of *B. bacilliformis*. This analysis led to the discovery of regions significantly rich in linear B-cell epitopes within the Prot_689 and Prot_504 proteins [34]. These proteins, which are non-homologous to those from other bacterial species associated with febrile illnesses, were subsequently expressed in the current study [34].

The expression of these potential antigenic proteins was conducted following a protocol described by a specialized Protein Production Research Facility [35] and oriented to determine the optimal conditions for maximizing yield and efficiency. The flashBAC ULTRA technology and Sf9 and High Five™ cells were utilized, with particular attention to temperature control and post-infection harvest timing, to enhance protein production outcomes. This approach facilitates a better understanding of antigenic protein expression and improves the diagnostic and therapeutic applications related to *B. bacilliformis*.

## 2. Materials and Methods

### 2.1. Recombinant Plasmids

**Figure A1:**
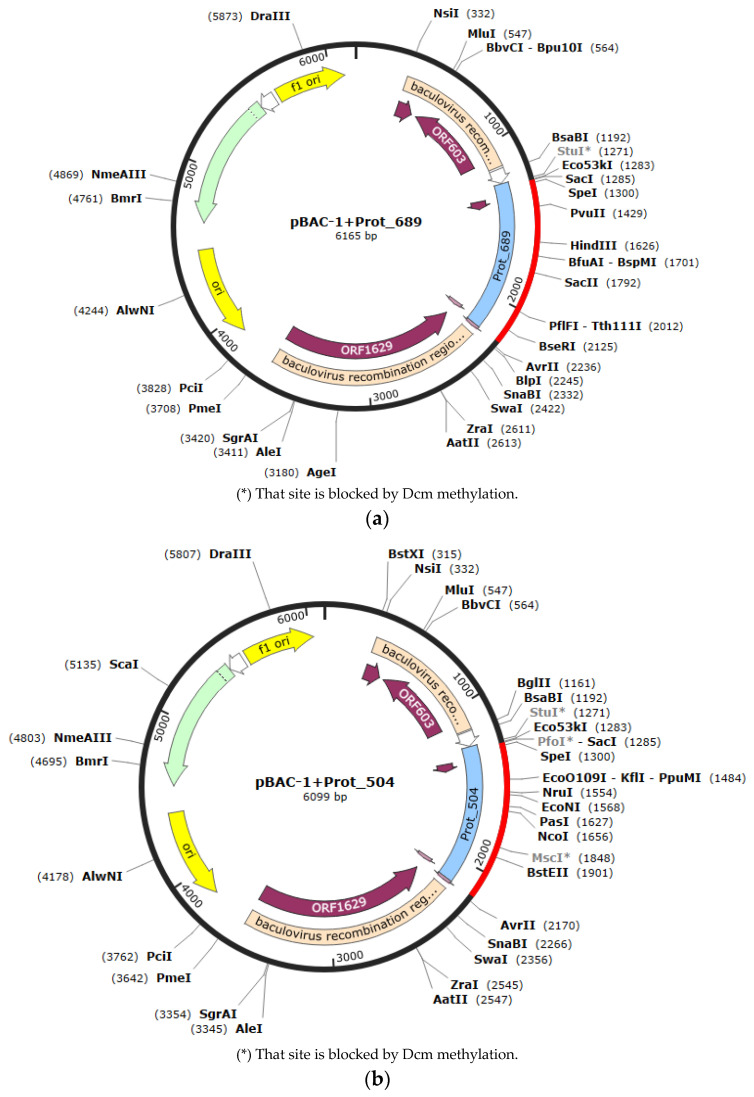
(**a**) Map of the pBAC-1+Prot_689 plasmid. (**b**) Map of the pBAC-1+Prot_504 plasmid. Both plasmids feature the gene of interest within the blue region.

Prior to this study, a comprehensive bioinformatics analysis was conducted by the LRNMEZOB to identify specific antigenic candidates for *B. bacilliformis*. Prot_689 and Prot_504 were selected to be expressed in the BEVS system. The plasmid was designed by the LRNMEZOB, and the plasmid synthesis of the two recombinant plasmids—pBac-1 (flashBAC ULTRA Technology)—containing sequences for specific antigenic candidates was carried out by the Protein Expression Facility at The University of Queensland (UQ-PEF). The design included the selection of a GP67 signal peptide, a sequence of interest, and a 10× histidine tag, all positioned under the control of the *polh* promoter. These sequences were subsequently cloned into the pBac-1 expression vector, resulting in the pBac-1+Prot_689 and pBac-1+Prot_504 constructs. These plasmids were preserved on filter paper until their resuspension. For a detailed visual representation of these genetic constructs, please refer to Appendix A, Figure A1.

### 2.2. Reactivation of Baculovirus Shuttle Vector

The One Shot OmniMAX 2 T1 Phage Resistant cells (Thermo Fisher Scientific, Waltham, MA, USA) were transformed with recombinant plasmids following the manufacturer protocol for plasmid propagation and maintenance. The transformed cells were then seeded onto Lennox Broth (LB) medium (Sigma-Aldrich, St. Louis, MO, USA), and incubated overnight at 37 °C until visible growth was observed by the turbidity of the culture medium. Subsequently, plasmid DNA was extracted using the PureYield Plasmid Miniprep System (Promega Corporation, Madison, WI, USA) and quantified for concentration. For confirmation of gene insertion, PCR analysis was conducted using two specific primers for the polyhedrin promoter (pH-F: 5′-GAT AAT TAA AAT GAT AAC CAT CTC GC-3′) and for the essential gene ORF1629 (1629-R: 5′-GCT CTA ACA TAC CAC CCT AAA G-3′).

The PCR components were as follows: buffer (1x), MgSO4 up to 2.0 mM, dNTP Mix (0.2 mM), primers pH-F and 1629-R (0.2 μM), and Platinum™ Taq DNA Polymerase (5 U/μL). The amplification program used for PCR analysis consisted of an initial denaturation step at 94 °C for 3 min. This was followed by 30 cycles of denaturation at 94 °C for 30 s, annealing at 60 °C for 30 s, and extension at 72 °C for 2 min. A final extension step was performed at 72 °C for 5 min to ensure complete amplification.

### 2.3. Recombinant Plasmid Sequencing and Characterization of Protein Sequences

The two plasmids were validated by Sanger sequencing and used for the generation of the recombinant baculoviruses. To confirm the accurate cloning of the Prot_689 and Prot_504 protein sequences into the pBac-1 vector, the recombinant plasmids were sequenced using specific primers for the polyhedrin promoter (primer pH-F) and for the essential gene ORF1629 (primer 1629-R) on an ABI 3500 genetic analyzer (Applied Biosystems, Foster City, CA, USA).

Additionally, the cloned nucleotide sequences of Prot_689 and Prot_504 were analyzed using SignalP (https://services.healthtech.dtu.dk/services/SignalP-6.0/ accessed on 19 July 2023), BLASTp (https://blast.ncbi.nlm.nih.gov/Blast.cgi?PROGRAM=blastp&PAGE_TYPE=BlastSearch&LINK_LOC=blasthome accessed on 19 July 2023), UniProt (https://www.uniprot.org/ accessed on 20 July 2023), and ProtParam (https://web.expasy.org/protparam/ accessed on 19 July 2023) to understand their signal peptides, sequence similarities, functions, and physicochemical properties, respectively. This analysis provided crucial insights into their biological functions and structural features, essential for optimizing the protein expression process and ensuring successful outcomes.

### 2.4. Insect Cell Culture Maintenance

The Sf9 and High Five™ insect cell lines were cultured in suspension using ESF 921™ Insect Cell Culture Medium, a protein-free medium (Expression Systems, Davis, CA, USA), maintained at 27 °C with continuous shaking at 135 rpm. It was crucial to ensure the suspension volume did not exceed 50% of the flask’s total volume for optimal aeration [36]. Cultures were initiated at cell densities of 1 × 10^6^ cells/mL for Sf9 and 3 × 10^5^ cells/mL for High Five™, with passages every other day to sustain health, monitored through trypan blue exclusion to ensure over 95% viability.

### 2.5. Evaluation of the Growth Curve

Growth curves of Sf9 and High Five™ cells were analyzed to identify the optimal phase (mid-logarithmic) for transfection and protein expression by seeding cells at 3 × 10^5^ cells/mL in ESF 921 medium and incubating at 27 °C with 135 rpm shaking for 10 days. This experiment was conducted in triplicate. Daily aliquots were used for cell counting and viability, with growth kinetics analyzed using GraphPad Prism (version 9.5.0).

### 2.6. Insect Cell Transfection

The process was conducted according to a detailed protocol [35]. Transfection of Sf9 cells involved recombination of the recombinant plasmid pBac-1, containing the sequence of interest, with the baculovirus genome (flashBAC ULTRA DNA), resulting in the production of recombinant baculovirus (P1-BV). Ensuring Sf9 cells were in the mid-logarithmic growth phase, as determined in the previous section, was a critical point. This way, 400 µL of Sf9 cells at 6 × 10^5^ cells/mL was seeded into 24-well plates and incubated at 27 °C for 60 min to allow cell adhesion. The transfection mix, prepared with 200 µL of Grace’s Insect Medium (Gibco, Thermo Fisher Scientific, Waltham, MA, USA), 20 ng of flashBAC ULTRA DNA (Oxford Expression Technologies, Oxford, UK), 100 ng of recombinant plasmid DNA (pBac-1+Prot_689 and pBac-1+Prot_504), and 1 µL of TransIT™ Insect Transfection Reagent (Mirus Bio LLC, Madison, WI, USA ), was incubated at room temperature for 30 min. This mix was then added to the Sf9 cell layer and incubated at 27 °C for 5 h. A control with cells only, without the transfection mix, was included. Afterward, ESF 921 medium supplemented with antibiotics and antimycotics was added, and the culture was incubated at 27 °C without shaking. On the fifth day, cells were monitored under an inverted light microscope for infection signs compared to the control. Seven days post-incubation, the supernatant containing the recombinant baculoviruses (P1-BV) was harvested.

### 2.7. Amplification of Viral Titer

The viral titer amplification (P2-BV) was performed in a 24-well deep-well plate by infecting 5 mL of Sf9 cells at 2 × 10^6^ cells/mL with 300 μL of the P1-BV harvest, followed by incubation at 27 °C with agitation in a humidified chamber. Total cell density, viable cell density, viability percentage, and average cell size were monitored at the endpoint. These parameters were assessed by taking an aliquot 4 days post-infection and using a hemacytometer. The culture was centrifuged at 1500 rpm for 5 min, and the supernatant containing the budding virus (P2-BV) was collected.

### 2.8. Verification of Baculovirus MOI

To ensure efficient protein expression in Sf9 cells, a plaque assay on P2-BV was conducted to verify that the multiplicity of infection (MOI) exceeded 1 [37]. The density of Sf9 cells was adjusted to 6 × 10^5^ cells/mL. These cells were then incubated with the P2-BV baculovirus and covered with a 1% agarose overlay. After a 7-day incubation period, the cell layers were stained with neutral red and further incubated for 24 h to facilitate the visibility of plaques. The MOI was calculated using the following formula [38]:

MOI (p.f.u. cells^−1^) = virus titer (p.f.u. ml^−1^) × ml of virus inoculum/total number of cells

### 2.9. Protein Expression Screening

Evaluations of protein production were conducted using Sf9 and High Five™ cell lines, seeded in a 24-well deep-well plate. Sf9 cells were cultured at a density of 3 × 10^6^ cells/mL, with each well containing 5 mL of culture, and were infected with 300 µL of P2-BV. High Five™ cells were cultured at a density of 1.5 × 10^6^ cells/mL, with each well also containing 5 mL of culture, but these were infected with 150 µL of P2-BV [35].

The cultures were incubated at either 27 °C or 21 °C with gentle agitation in a humidified incubator. Samples were collected at 48 and 72 h post-infection for the 27 °C condition and at 96 and 120 h post-infection for the 21 °C condition. Subsequently, the aliquots were centrifuged at 13,000 rpm for 5 min to facilitate the separation of the components. This process resulted in a clear demarcation between the cell pellet, which contained intracellular proteins, and the supernatant, which held the extracellular proteins. These assessments aimed to identify and select the optimal conditions for the highest yield of recombinant protein production.

### 2.10. SDS-PAGE and Western Blot

The samples were processed to separate the proteins by SDS-PAGE and subsequently identify them through Western blotting. The cellular pellet fraction was sonicated using an ultrasonic sonicator (3 cycles of 20 s at 50% amplitude) in lysis buffer (20 mM monosodium phosphate, 500 mM NaCl, 20 mM imidazole, 1% Triton X-100, 2 mM MgCl_2_, and protease inhibitor, pH = 7.5). Quantification of both the supernatant and the pre-lysed cellular pellet fractions was performed using the Bradford method and treated with a reducing buffer containing Tris/HCl pH 6.8, 5% 2-mercaptoethanol, and 2% SDS. The treated cellular pellet samples were loaded at the same concentration onto 15% polyacrylamide gels, while the supernatants were loaded in equal volumes. Electrophoresis was conducted at 80 V for a 10 min pre-run and at 160 V for 1 h. After electrophoresis, the SDS-PAGE gel was stained with Coomassie blue R-250 to visualize the protein bands. Subsequently, the Western blot analysis was carried out using a gel prepared under the same parameters as the SDS-PAGE. Thus, the proteins were transferred from the gel to a nitrocellulose membrane using the wet Western blot transfer system for 1 h at 60 V. The membranes were blocked for 90 min with PBS-Tween and 5% skim milk. Since the proteins possess a histidine tag, probing was conducted using 0.5 µg/mL of His tag polyclonal antibody, HRP (Invitrogen), at 37 °C for 1 h. After probing, the membranes were washed five times with PBS-Tween, each wash lasting 5 min with agitation. Finally, TMB solution for blotting was added to reveal the bands.

### 2.11. Data Analysis: Densitometry

Band visualization of SDS gel was performed using a ChemiDoc XRS+ Imaging System (BIO-RAD), and the Western blot results were subjected to densitometric analysis using Image Lab 6.1 software. This analysis aimed to compare the expression of equal volumetric samples collected under varying conditions, including different temperatures, post-infection time points, and cell lines Sf9 and High Five™. The analysis involved comparing the relative intensity of the bands, with the band exhibiting the highest intensity serving as the normalization reference. Band selection was predicated on the anticipated molecular weight. The results, presented as percentages, indicate which conditions yielded the optimal conditions for scaling up protein expression. 

### 2.12. Amplification of P2-BV Passage

To scale up protein expression, it is essential to produce a larger volume of P2-BV [37]. A 50 mL culture of Sf9 cells at an initial density of 1 × 10^6^ cells/mL was used, and 25 µL of P2-BV was added. Incubation was carried out at 27 °C with shaking at 120 rpm. After three days, signs of infection were observed, including cessation of growth, increased cell size, and decreased viability. Subsequently, the culture was centrifuged at 400 × *g* for five minutes to separate the supernatant containing the high concentration of the amplified virus, which would be used to scale up protein expression. 

### 2.13. Scale-Up of Protein Expression

The scale-up process was implemented following parameters that, as determined by densitometry during the screening phase, enabled the highest yield of recombinant protein expression. The expression volume was increased to 50 mL, utilizing High Five™ cells at a density of 1.5 × 10^6^ cells/mL. To this mixture, 750 µL of amplified P2-BV was added, and the culture was incubated at the optimal temperature identified earlier. To ensure the effectiveness of the scale-up, the expression flask was monitored one day prior to the predetermined harvest time. The sample collection was timed to coincide with the peak expression yield, in line with the densitometry results from the screening phase. Following this, the culture was harvested and centrifuged, and the pellet was isolated for subsequent processing.

### 2.14. Purification Test

Protein purification was conducted using Gravity-Flow Immobilized Metal Affinity Chromatography (IMAC). This technique offers selectivity, simplicity, and accessibility. Initially, clarified cell lysates were sonicated in lysis buffer and then applied to a column packed with Nuvia^TM^ IMAC Ni-Charged Resin (BIO-RAD, Hercules, CA, USA). The protein binding was conducted using a buffer containing 20 mM imidazole, 500 mM NaCl, and 20 mM sodium phosphate. To remove non-specifically bound proteins, the column was washed twice with a buffer containing 50 mM imidazole, 500 mM NaCl, and 20 mM sodium phosphate. Subsequently, proteins of interest, marked by their histidine tags, were eluted using a buffer with a higher concentration of imidazole (700 mM), 500 mM NaCl, and 20 mM sodium phosphate designed to disrupt the interaction between the His-tag and the nickel ions on the resin. All elution aliquots were subjected to protein quantification using the Bradford assay, and the fraction with the highest concentration was selected. Later, the specificity of the purification method was confirmed through densitometry after SDS-PAGE and Western blot analyses.

### 2.15. Seroreactivity Assessment

Recombinant proteins Prot_689 and Prot_504 were assessed for seroreactivity when expressed in insect cells, thereby confirming their identity as the antigenic proteins intended for expression. This evaluation utilized reference serum from patients with positive diagnostics for Carrion’s disease, positive serum for other etiologies (such as brucellosis), and a negative control serum from a healthy patient provided by LRNMEZOB. Western blot membranes were prepared and cut into strips. These strips were then incubated with 1/50 dilutions of both the disease-specific and control sera at room temperature with agitation for 15 min, followed by a 45 min incubation at 37 °C. After five washes with PBS-Tween, the strips were treated with a 1/1000 dilution of Goat anti-Human IgG Whole-molecule HRP conjugate (Sigma, Tokyo, Japan) at 37 °C for 1 h. Visualization of the reaction between the protein and serum antibodies was achieved using TMB solution for blotting, aiming to identify specific antibodies that recognize Prot_689 and Prot_504.

## 3. Results

### 3.1. Verification and Validation of Gene Insertion into pBAC-1 Plasmid

To ensure the successful expression of target proteins in insect cells, the pBAC-1 vector plasmid, harboring the genes of interest Prot_689 and Prot_504, was analyzed.

Table 1 shows the theoretical calculations to estimate the molecular weight based on the number of amino acids, the number of bases, and the resulting base pairs. These foundational data set the stage for anticipating the size of both the insert and the complete plasmid.

SnapGene 7.2 software was employed to simulate PCR reactions using primers pH-F and 1629-R. The results confirmed the inclusion of the polyhedrin promoter, the signal peptide, the gene of interest, the histidine tag, and ORF1629 within the constructs.

**Figure 1:**
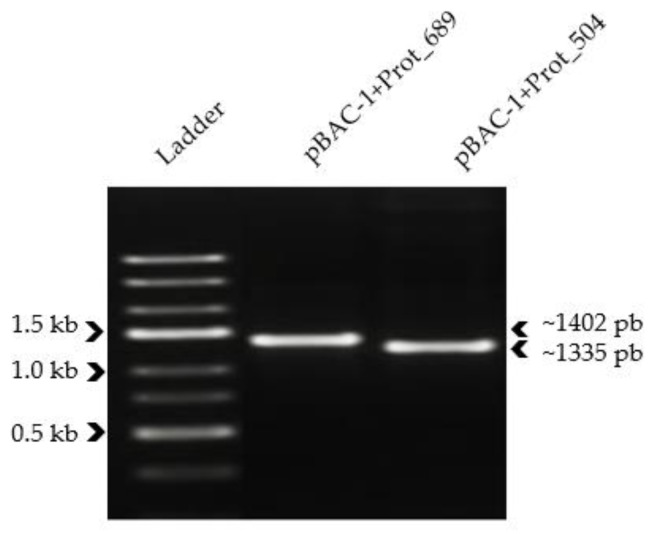
PCR analysis of recombinant plasmids. Image of bands obtained on 1.8% agarose gel for the visualization of PCR products.

The integrity of the recombinant plasmid and the insert was further observed through gel electrophoresis. Figure 1 captures the gel image, showcasing unique and intense bands corresponding to the anticipated sizes. Lane 2 reveals the amplification product of pBac-1+Prot_689 at approximately 1402 bp, while lane 3 displays pBac-1+Prot_504 at about 1335 bp. The band sizes observed in the electrophoresis closely approximate the theoretical sizes calculated, as detailed in Table 1, thereby confirming the presence of the inserted gene within both plasmid constructs.

Sanger sequencing served as the tool for sequence validation. The sequences of pBac-1+Prot_689 and pBac-1+Prot_504 were analyzed, revealing no mutations and confirming the correct reading frame. The high-quality sequencing reinforced the reliability and successful cloning of the genes of interest into the pBAC-1 vector, setting the stage for subsequent expression analysis.

### 3.2. Identification and Functional Analysis of Proteins

#### 3.2.1. Prot_689: RlpA

**Figure 2:**
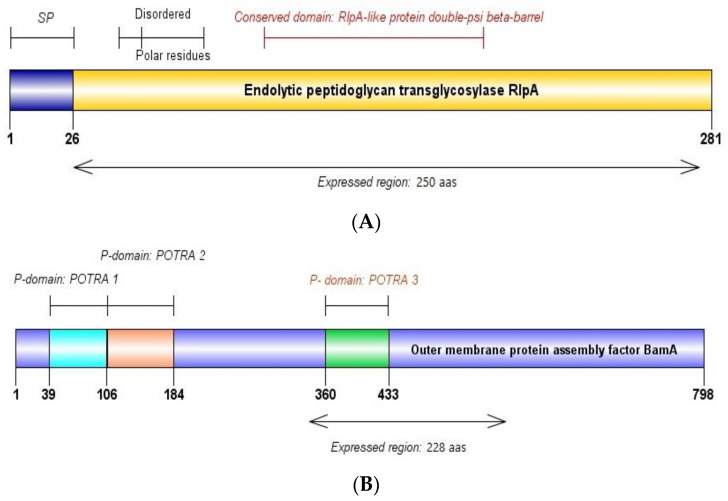
Domain mapping and expressed regions of recombinant proteins (**A**) RlpA and (**B**) BamA. SP: signal peptide. RlpA: Rare lipoprotein A. POTRA: Polypeptide Transport-Associated. aas: amino acids.

Blastp analysis reveals that Prot_689 shares 91.7% identity with *Bartonella bacilliformis*’ septal ring lytic transglycosylase RlpA family protein, accession WP_005767158.1. This protein contains a Rare lipoprotein A domain with peptidoglycan hydrolase activity targeting “naked” glycans and a C-terminal SPOR domain associated with cell wall/membrane/envelope biogenesis. SignalP predicts a signal peptide spanning amino acids 1–38 with a likelihood score of 0.7615, identified as gp67, guiding the protein to its cellular membrane localization. UniProt indicates its localization to the cellular membrane and features a disordered region, compositional bias, and a double-psi beta-barrel domain characteristic of RlpA-like proteins (Figure 2A), with post-translational modifications including lipoprotein palmitoylation. The ProtParam analysis shows the protein comprises 264 amino acids, with a processed form molecular weight of 29.8 kDa and an unprocessed form of 33.8 kDa. A theoretical isoelectric point (pI) of 9.57 classifies the protein as basic. It contains high proportions of lysine (12.9%), serine (8.3%), and leucine (7.6%). The protein’s half-life varies by cell type, indicating differential stability across organisms. An instability index of 25.52 categorizes the protein as stable. An aliphatic index of 78.30 suggests a high proportion of aliphatic amino acids, and a GRAVY value of −0.627 denotes that the protein is predominantly hydrophilic.

#### 3.2.2. Prot_504: BamA

Our bioinformatic analysis reveals that Prot_504 exhibits a 94.29% identity with the outer membrane protein assembly factor BamA from *Bartonella bacilliformis* (accession WP_005766757.1), as determined by the Blastp analysis. SignalP identifies a signal peptide spanning amino acids 1–38 with a score of 0.994, designated as gp67, essential for the protein’s subcellular localization to the outer membrane. According to UniProt, this outer membrane protein assembly factor BamA is part of the complex involved in the assembly and insertion of beta-barrel proteins into the outer membrane. The protein contains three POTRA domains (Figure 2B), as per the InterPro annotation. The ProtParam analysis indicates the protein comprises 242 amino acids. The processed protein has a molecular weight of 27.3 kDa, while the unprocessed form is 31.4 kDa, with a theoretical pI of 5.75. The amino acid composition includes a high content of glycine (9.9%) and serine (9.5%), with arginine content at 7.9% and lysine at 1.7%. The protein lacks tryptophan and cysteine. An instability index of 37.07 classifies the protein as stable. The aliphatic index is 70.04, and a GRAVY of −0.659 indicates the protein’s hydrophilicity.

### 3.3. Growth Curve Assessment of Insect Cells

As depicted in Figure S1, the mid-logarithmic phase for Sf9 cells occurs on the 4th day, and according to Table S1, the average doubling time is approximately 48.79 h. For High Five™ cells, the mid-logarithmic phase is reached on the 3rd day (Figure S2), with an average doubling time of 23.27 h, as shown in Table S2. Since cell transfection is performed during the mid-logarithmic phase, these data will be utilized to produce P1-baculovirus (P1-BV) and screening expression proteins (P3-BV).

### 3.4. Insect Cell Transfection P1-BV and Amplification of Viral Titer P2-BV

In the process of optimizing conditions for baculovirus vector production, Sf9 cells were used for transfection and subsequent viral titer amplification. P2-BV was generated through transfection followed by amplification to yield P1-BV.

**Figure 3:**
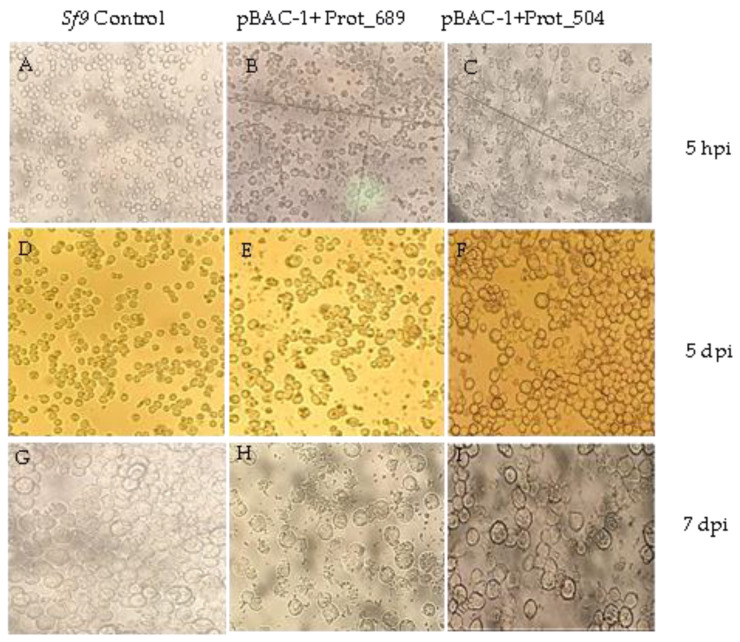
Temporal analysis of Sf9 cell response to transfection. (**A**) Sf9 control cells at 5 h post-infection (hpi). (**B**,**C**) Transfected Sf9 cells at 5 hpi. (**D**) Sf9 control cells 5 days after the assay initiation. (**E**,**F**) Transfected Sf9 cells at 5 days post-infection (dpi). (**G**) Sf9 control cells 7 days after the assay initiation. (**H**,**I**) Transfected Sf9 cells at 7 dpi. Observed with a 40× inverted microscope.

A microscopic examination revealed distinct cellular responses to transfection compared to the Sf9 control. Notably, at 5 h post-transfection, the transfected cells displayed granulation and lysis (Figure 3B,C). Up to 5 days post-initiation, the transfected cells exhibited increased size, granulation, and debris accumulation, along with a reduction in overall cell growth at the same time point (Figure 3E,F). By the seventh day, while the control cells demonstrated significant proliferation (Figure 3G), the transfected cells were marked by a decrease in density, enlarged cell diameters, and evident granulation (Figure 3H,I).

In relation to this, the results of the amplification of viral titer, cellular density, and viability metrics post-P2-BV infection indicated a drop in viability from an initial 95% to 20–30% for both the control and transfected cells (Table S3). The amplification process began with a cell density of 2 × 10^6^ cells/mL, with the infected cells ultimately achieving a higher density compared to the controls, which recorded the lowest density at 4.07 × 10^5^ cells/mL (Table S3).

### 3.5. Verification of MOI

This verification confirmed that, following the established protocol, the MOI for Prot_689 expression in Sf9 is 3, derived from the recommended P2-BV inoculum volume. Similarly, for Prot_504 expression in this cell line, the protocol yields an MOI of 2.5. This step of confirming the MOI is essential, serving as a critical control point in the process of recombinant protein production and ensuring the MOI is neither too low nor too high for optimal expression.

### 3.6. Selection of Optimal Conditions for Protein Expression 

**Figure 4:**
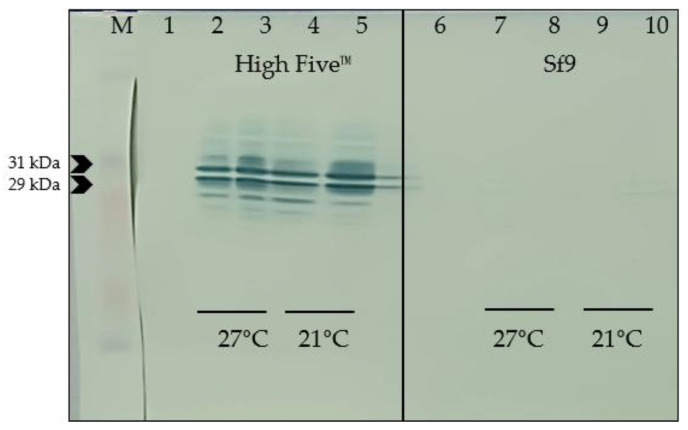
Higher expression of recombinant protein Prot_689 in High Five™ cells compared to Sf9: Western blot analysis of cellular pellet fractions using anti-His tag HRP-conjugated antibody.

**Figure 5:**
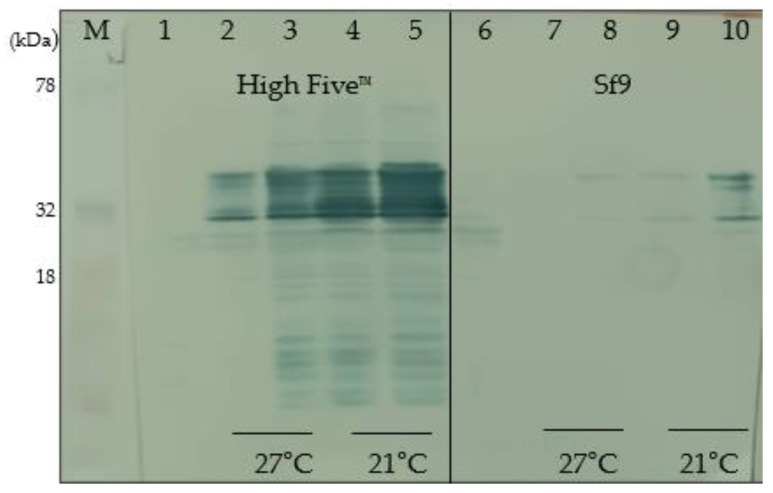
Comparative expression of recombinant protein Prot_504 in High Five™ and Sf9 cells: Western blot analysis of cellular pellet fractions using anti-His tag HRP-conjugated antibody.

While SDS-PAGE does not provide clear evidence of recombinant protein expression, Western blot analysis using the His tag polyclonal antibody HRP (Invitrogen, Waltham, MA, USA) successfully demonstrated the presence of the target proteins. The Western blot images, as shown in Figure 4 and Figure 5, visually confirm that both Prot_689 and Prot_504 achieved their highest expression levels under conditions of High Five^TM^ cells at 21 °C and harvested at 120 hpi (Table 2). Notably, no bands were observed in the Western blot of the supernatants, indicating that the recombinant proteins were detected in the pellet fractions. This was consistently observed for both proteins, highlighting the efficacy of these specific conditions for optimal protein expression.

Further supporting these observations, densitometry analysis (Tables S4 and S5) reinforces the visual evidence provided by the Western blots and quantitatively demonstrates that this condition yields the highest expression levels, as depicted in the summary graph and representative graph with percentages. The densitometry data, detailed in the Supplementary Tables, align with the visual patterns observed in the figures, underscoring the superior performance of High Five^TM^ cells at 21 °C and 120 hpi for the expression of Prot_689 and Prot_504 (Table 3). This comprehensive analysis has been instrumental in establishing effective expression, scale-up expression, and purification protocols, ensuring high-quality protein yields for further downstream applications.

### 3.7. Purification Testing of Scale-Up Protein Expression

In the Supplementary Materials, Figures S3 and S4 and Table S6 illustrate the selection process for the eluate fractions of Prot_689 and Prot_504 post-purification with the highest concentration of purified protein. This selection was critical for ensuring that subsequent analyses, including SDS-PAGE and Western blot, were performed on samples with the most significant enrichment of the target proteins.

**Figure 6:**
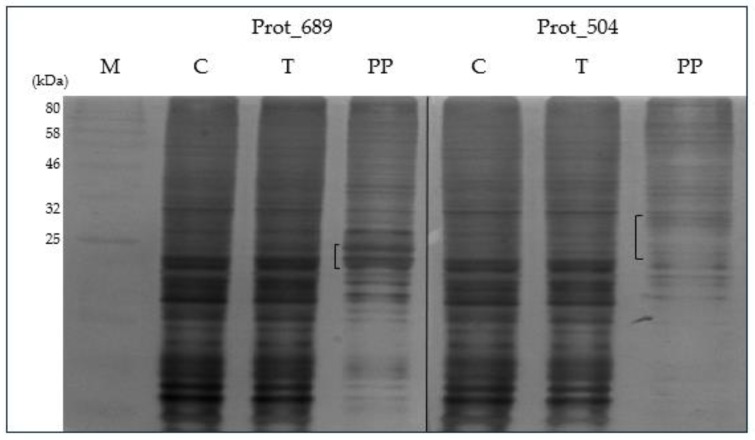
SDS-PAGE analysis of purified pellet fractions of Prot_689 and Prot_504 expressed in High Five™ cells: polyacrylamide gel, 15% stained with Coomassie blue R-250. M: protein marker. C: High Five™ cell control. T: total protein. PP: protein post-purification.

**Figure 7:**
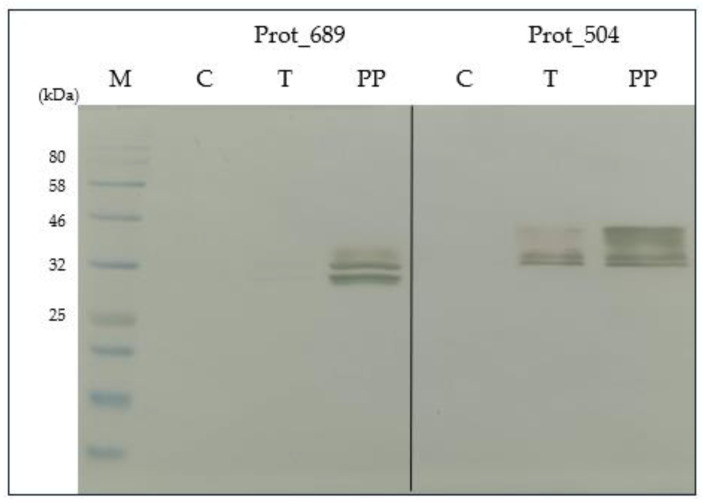
Western blot analysis of purified pellet fractions of Prot_689 and Prot_504 expressed in High Five™ cells: specific detection with anti-His tag antibody. M: protein marker. C: High Five™ cell control. T: total protein. PP: protein post-purification.

SDS-PAGE (Figure 6) and Western blot analysis (Figure 7), confirmed the presence of the Prot_689 and Prot_504 target proteins post-purification, with significant bands aligning with the expected molecular weights of these recombinant proteins. The purification process effectively reduced non-specific bands prevalent in the control cell lysates. For Prot_689, this resulted in the elimination of several bands corresponding to other proteins, revealing two distinct bands absent in the control. These bands, with molecular weights of approximately 29 kDa and 31 kDa, closely aligning to the theoretical weights, are indicative of the processed (without signal peptide) and unprocessed (including signal peptide) forms of the protein, respectively. Regarding Prot_504, the Western blot analysis was particularly revealing, showing three distinct bands consistent with weights higher than the theoretical molecular weight in both the total and purified protein samples, which could indicate post-translational modifications.

The identification of potential post-translational modifications is important to explain the different weights of proteins obtained. Experiments on seroreactivity will be crucial to determining whether the obtained proteins represent active or functional forms of Prot_689 and Prot_504.

### 3.8. Seroreactivity Assessment of Prot_689 and Prot_504

**Figure 8:**
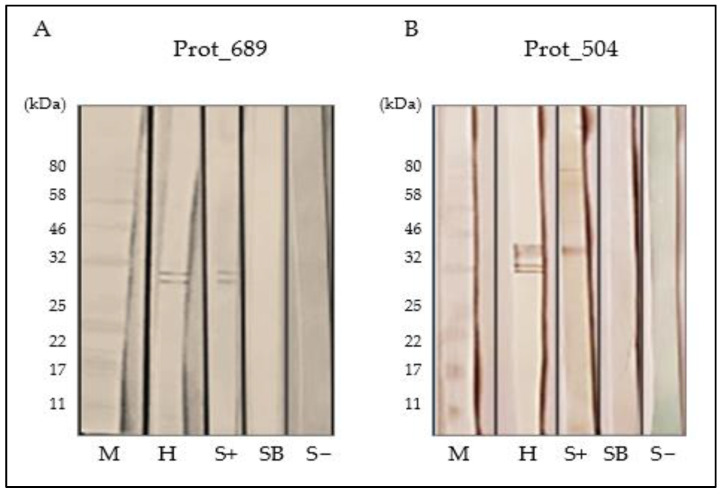
Evaluation of seroreactivity using Western blot: nitrocellulose membranes impregnated with (**A**) Prot_689 and (**B**) Prot_504 for assessing immunological activity of selected proteins. M: protein marker. H: anti-His tag antibody. S+: human serum positive for Carrion‘s disease. SB: serum positive for brucellosis. S−: negative human serum.

As depicted in Figure 8, antigenic strips were challenged with reference serum from a patient with a positive diagnosis for Carrion’s disease (S+), sera positive for other etiologies (SB) (such as brucellosis), and a negative control serum (SB−) from a healthy patient provided by the LRNMEZOB. The proteins demonstrated seroreactivity with the positive serum (S+), resulting in bands that corresponded to the same bands obtained in reactions with the anti-His tag (H) antibody. Specifically, for Prot_689, the same two bands were observed at 29 kDa and 31 kDa, with no cross-reaction observed with the brucellosis serum (SB) or with the negative serum (S−). For Prot_504, using the reference serum (S+), a prominent band close to 40 kDa was evident, with two lighter bands of lower molecular weight not being discernible, unlike the reaction with the anti-His tag antibody (H). Neither the brucellosis patient serum (SB) nor the negative control serum (S−) exhibited any reactive bands.

## 4. Discussion

In this study, we have explored the expression of two *Bartonella bacilliformis* recombinant proteins, Prot_689 and Prot_504, using the Baculovirus Expression Vector System (BEVS). Our main goal was to optimize expression parameters such as insect cell lines, temperature, and harvest time to enhance the yield of these potential antigenic proteins, aiming to improve diagnostic methods for Carrion’s disease. 

The optimization of expression conditions was a notable focus of our research, as robust and efficient upstream processes were necessary [37]. Western blot analysis, supported by densitometry, showed that the highest expression levels of Prot_689 and Prot_504 were achieved in High Five™ cells (Table 3). 

At 27 °C, the maximal yield was observed at 72 hpi (Figure 4 and Figure 5), which is consistent with the optimal window for recombinant protein expression in the BEVS system [39], where late gene expression under the control of the polyhedrin promoter peaks. However, studies have evaluated that proteins expressed in High Five™ cells suffer more proteolysis, which can sometimes be mitigated by lowering the incubation temperature from 27 °C to 21 °C [40]. 

Contrary to the common belief that higher temperatures facilitate greater protein expression, our results indicate that High Five™ cells at 21 °C yield higher protein amounts of Prot_689 and Prot_504 when harvested at 120 hpi. This aligns with reports that lower cultivation temperatures (20–25 °C) can increase other eukaryotic systems’ product titers by reducing folding stress, thus facilitating correct protein folding and extending the period for more efficient folding and substantial protein accumulation [41]. However, the effectiveness of this approach depends on the specific characteristics of the heterologous protein [41]. High Five™ cells have emerged as more effective for recombinant protein expression, suggesting their suitability for producing difficult-to-express proteins [33,36,42,43]. 

At this point, the protein expression parameters have been defined. UniProt describes a functional analysis of Prot_689 as a septal ring lytic transglycosylase RlpA family protein involved in peptidoglycan architecture during cell division. The activity of RlpA is regulated on at least two levels—by the SPOR domain, which recruits the protein to the septal ring, and by the amidases, whose activity is regulated by a host of septal ring-associated proteins [44]. Studies in *Pseudomonas aeruginosa* have shown that RlpA is necessary for efficient daughter cell separation and rod-shape maintenance [44].

Prot_504, identified as BamA, is essential for assembling outer membrane proteins (OMPs) in gram-negative bacteria, highlighting its potential as a diagnostic antigen candidate [34]. This protein contains a POTRA domain, which is involved in protein–protein interactions and the nucleation of β-strand formation in nascent outer membrane proteins (OMPs) [45]. The folding and integration of OMPs into the outer membrane depend on the Bam complex, a universally conserved mechanism [46,47]. The expression of BamA in insect cells poses challenges due to the lack of specific bacterial chaperones and the Bam complex required for its proper folding, which protects them from aggregation and facilitates membrane insertion [47]. 

Choosing an appropriate expression protein system for bacterial outer membrane proteins (OMPs) like BamA is challenging due to their complex folding and assembly needs. While *E. coli* is adequate for smaller membranes, eukaryotic systems, such as insect cells, are often favored for their ability to perform necessary post-translational modifications for more complex proteins [48]. 

Regarding the scale-up process, if increased protein yield or frequent production cycles are required, the BEVS is a guaranteed choice [43]. Scaling up protein expression to a volume of 50 mL was manageable. The purification tests using Gravity-Flow Immobilized Metal Affinity Chromatography, followed by SDS-PAGE, indicated that while the purification was not completely efficient, it reduced contaminants, as seen in Figure 6. These results suggest that while this method is adequate for preliminary studies, alternative purification strategies could be employed for applications requiring larger volumes, structural characterization, or the development of diagnostic tests for patient use.

Western blot analysis of His-tagged purified fractions detected the Prot_689 and Prot_504 target proteins and revealed multiple bands. In the case of Prot_689, the two bands present have weights similar to the theoretical values corresponding to the processed (without signal peptide) and unprocessed (with signal peptide) forms. This is consistent with the protein being retained intracellularly, as reinforced by the results. Despite using a signal peptide for secretion to facilitate the recovery of recombinant proteins, most of the protein was found in the cell pellet, and the secreted protein in the supernatant was not detectable). This indicates that the signal peptide did not function as expected for efficient secretion, and the majority of the recombinant protein remained within the cells, which aligns with other studies showing similar retention issues with signal peptides in insect cells [41,49]. For Prot_504, the three bands (Figure 7) with weights higher than the theoretical weight can be attributed to insect cells performing post-translational modifications (PTMs), particularly in glycosylation processes, which increase the molecular weight [50,51]. While *Bartonella bacilliformis* is a prokaryotic pathogen, expressing its proteins in a eukaryotic system like insect cells allows for the addition of PTMs. N-glycosylation in insect cells tends to be the most common PTM, less complex and more homogeneous, impacting the protein’s function, proper folding, stability, and immunogenicity, potentially altering its biological properties [51,52]. The evaluation using NetNGlyc 1.0 (https://services.healthtech.dtu.dk/services/NetNGlyc-1.0/ (accessed on 6 January 2024) predicted N-glycosylation sites in Prot_504 (Figure S5), indicating potential glycosylation modifications when expressed in insect cells, which could contribute to and explain their observed heterogeneity in the SDS-PAGE and Western blot analyses (Figure 6 and Figure 7).

To assess whether these potential modifications and possible processed and unprocessed forms affected the immunogenic activity of the expressed proteins, a seroreactivity assay was conducted using reference human sera with a positive diagnosis for Carrion’s disease (S+). The results indicate a clear profile. For Prot_689, the presence of two bands in bartonellosis-positive serum and the absence of reactivity in negative sera suggest that antibodies in human serum are highly specific for its epitopes, indicating a specific immune reaction that could be harnessed for diagnostic or therapeutic applications.

For Prot_504, human serum exclusively recognized the higher-molecular-weight band, which likely has a post-translational modification. The lack of reactivity towards lower-weight bands may be due to these epitopes not being present or being misfolded in the smaller forms of the protein, or to the specific post-translational modification required for antibody binding only being present in the higher-weight band. This indicates that only the higher-molecular-weight form of Prot_504 possesses the correct conformation and/or post-translational modifications to be recognized by the human immune system.

## 5. Conclusions

In conclusion, our study significantly contributes to the field of recombinant protein expression by identifying key parameters for the successful protein yield of two *Bartonella bacilliformis* proteins. The insights provided by Adeniyi and Lua (2020) [37] were effectively applied in the LRNMEZOB, constituting a pilot study that establishes the appropriate parameters to develop a recombinant protein expression platform with antigenic potential. This offers a pathway to improved diagnostic methodologies that could enhance disease management and control and improve the timely diagnosis of neglected vector-borne bacterial diseases. It is recommended that future studies focus on further functional validations and assessing the efficacy of these proteins in clinical diagnostic settings, particularly Prot_689, which demonstrated antigenic potential by reacting specifically to human sera.

## Figures and Tables

**Table 1:** Characterization of recombinant plasmids.

Elements of Constructs	pBAC-1+Prot_689	pBAC-1+Prot_504
Protein of interest’s sequence (nucleotides)	932	865
Signal peptide (38 amino acids)	114	114
Restriction sites (4)	24	24
10× His-tag	30	30
pBac-1 plasmid backbone (pH+1629)	302	302
Approximate band size to identify (bp)	1402	1335

**Table 2:** Expression conditions of Prot_689 (Figure 4) and Prot_504 (Figure 5).

Lane	Cell Line	Temp.	Harvest Time	Fraction
M	Protein marker			
1	High Five™—control	27 °C	72 hpi	Pellet
2	High Five™	27 °C	48 hpi	Pellet
3	High Five™	27 °C	72 hpi	Pellet
4	High Five™	21 °C	96 hpi	Pellet
5	High Five™	21 °C	120 hpi	Pellet
6	Sf9—control	27 °C	72 hpi	Pellet
7	Sf9	27 °C	48 hpi	Pellet
8	Sf9	27 °C	72 hpi	Pellet
9	Sf9	21 °C	96 hpi	Pellet
10	Sf9	21 °C	120 hpi	Pellet

**Table 3:** Effects of cell line, temperature, and harvest time on the expression of proteins 689 and 504.

			Relative Quantity			
Cell Line	T°	Harvest Time	Prot_689		Prot_504	
High Five™	27 °C	48 hpi	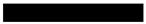	46.6%	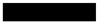	36.6%
		72 hpi	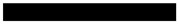	56.8%	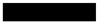	36.8%
	21 °C	96 hpi	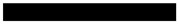	56.9%	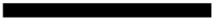	80.0%
		120 hpi	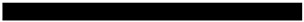	100.0%	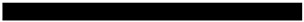	100.0%
Sf9	27 °C	48 hpi		0.0%		0.0%
		72 hpi	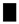	3.0%	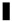	2.8%
	21 °C	96 hpi		0.0%	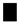	3.1%
		120 hpi	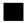	4.0%	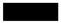	25.9%

The size of the bars represents the intensity of the WB band normalized to the highest intensity at 100%.

## Data Availability

Data are contained within the article and Supplementary Materials.

## Supplementary Materials

The following supporting information can be downloaded at https://www.mdpi.com/article/10.3390/pathogens13080690/s1: Figure S1: Growth and Viability of Sf9 Cells: Time vs. Log Cell Density; Figure S2: Growth and Viability of High Five™ Cells: Time vs. Log Cell Density; Figure S3: Standard Curve BSA of Quantification of Purified Proteins. Figure S4: Purification Fractions Collected and Concentration Profiles. Figure S5: Predicted N-glycosylation Sites in Prot_504 by NetNGlyc 1.0. Table S1: Growth kinetics results of Sf9 cells using GraphPad Prism (9.5.0); Table S2: Growth kinetics results of High Five™ cells using GraphPad Prism (9.5.0). Table S3: Cell Viability and Density Results of P2-BV. Table S4: Densitometry Analysis of Prot_689. Table S5: Densitometry Analysis of Prot_504. Table S6: Quantification of Purified Proteins Concentration by the Bradford Method.
