# Supplementary material for: Baculovirus-Assisted Production of Bartonella bacilliformis Proteins: A Potential Strategy for Improving Serological Diagnosis of Carrion’s Disease
Source: Pathogens. 2024 Aug 15;13(8):690. doi: 10.3390/pathogens13080690 (PMC11357310; doi:10.3390/pathogens13080690)
Supplement: Supplementary file 1 [file pathogens-13-00690-s001.zip › pathogens-2979587-supplementary.pdf]

Supplementary Materials

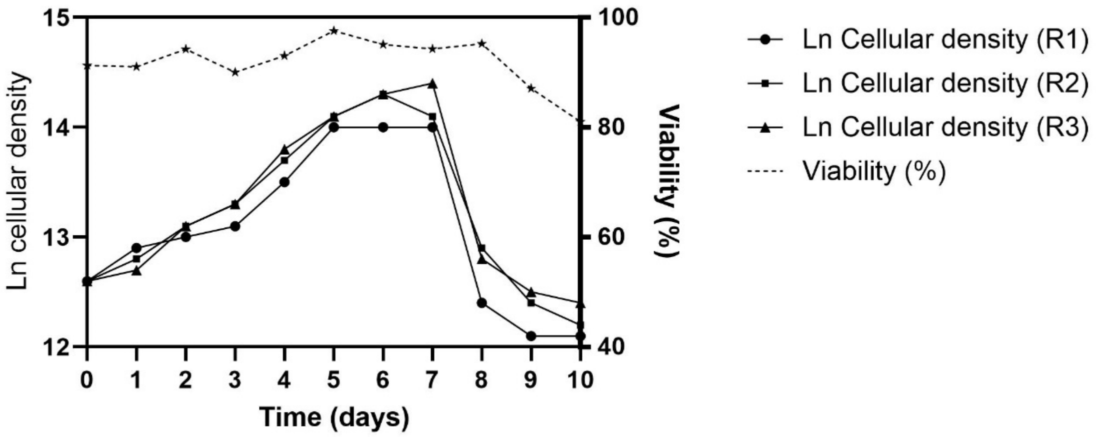

Figure S1. Growth and Viability of Sf9 Cells: Time vs. Log Cell Density.

Table S1. Growth kinetics results of Sf9 cells using GraphPad Prism (9.5.0)

**Exponential growth with log(population)**			
**Best-fit values**	**Sf9 (R1)**	**Sf9(R2)**	**Sf9(R3)**
logY0	12.24	12.36	12.31
k	0.32	0.33	0.36
Doubling time (hours)	50.73	49.44	46.2

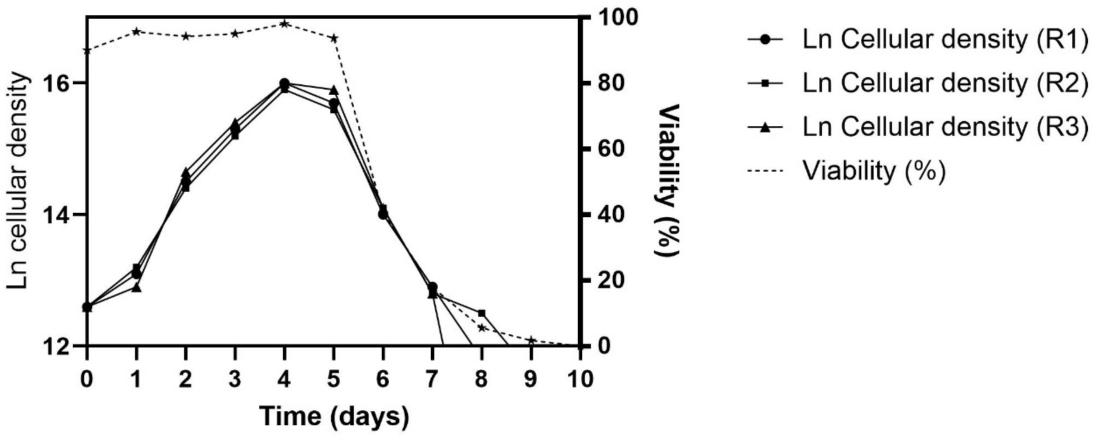

Figure S2. Growth and Viability of High Five™ Cells: Time vs. Log Cell Density

Table S2. Growth kinetics results of High Five™ cells using GraphPad Prism (9.5.0)

**Exponential growth with log(population)**			
**Best-fit values**	**High Five™ (R1)**	**High Five™ (R2)**	**High Five™ (R3)**
logY0	12.74	12.78	12.69
k	0.72	0.68	0.75
Doubling time (hours)	23.13	24.58	22.11

Table S3. Cell Viability and Density Results of P2-BV

	Initial conditions	4 days post-infection		
		Control	PEF 2023-2235	PEF 2023-2236
Cellular viability*	95%	23.24%	29.56%	31.02%
Cellular density*	2.0×10^6	4.07×10^5	4.46×10^5	4.76×10^5

* The data correspond to the average of four repetitions

Table S4. Densitometry Analysis of Prot_689

Lane	Band No.	Mol. Wt. (KDa)	Relative Front	Adj. Volume (Int)	Volume (Int)	Rel. Quant.
1	1	216	0.00	12675	2854605	0.00
1	2	132	0.08	1176565	20860905	0.03
1	3	78	0.14	5666570	42501550	0.14
1	4	45.7	0.41	5736510	42533985	0.15
1	5	32.5	0.53	2300870	41139930	0.06
1	6	18.4	0.83	4034355	46460765	0.10
1	7	7.6	1.00	2456545	11442665	0.06
3	1	44.73	0.42	15921035	36990915	0.41
3	2	40.93	0.45	15282280	28475590	0.39
3	3	35.35	0.50	4534725	23186475	0.12
4	1	44.84	0.42	23876072	36889380	0.61
4	2	40.84	0.45	23624392	35843544	0.60
4	3	35.27	0.50	6988080	20296452	0.18
5	1	44.44	0.42	27339195	41272725	0.70
5	2	40.66	0.45	23041655	33959380	0.59
5	3	34.96	0.51	12849655	30221100	0.33
6	1	44.76	0.42	35616750	50237460	0.91
6	2	40.76	0.45	39299065	55779815	1.00
6	3	35.19	0.50	6749405	22864140	0.17
11	1	47.47	0.39	159552	4298112	0.03
11	2	44.14	0.42	1024640	15023104	0.04

Table S5. Densitometry Analysis of Prot_504

Lane	Band No.	Mol. Wt. (KDa)	Relative Front	Adj. Volume (Int)	Volume (Int)	Rel. Quant.
1	1	132.00	0.09	5043168	40189110	0.09
1	2	78.00	0.15	9828702	60850140	0.18
1	3	45.70	0.42	15222324	76826334	0.29
1	4	32.50	0.97	3831204	68319264	0.07
3	1	53.17	0.34	19787560	43295655	0.37
3	2	51.08	0.36	9623770	23691005	0.18
3	3	49.41	0.38	2899260	18180045	0.05
3	4	45.58	0.42	13313300	40113385	0.25
3	5	45.2	0.43	19491225	41078635	0.37
3	6	44.14	0.47	1828320	13510380	0.03
4	1	61.60	0.27	505635	7196020	0.01
4	2	53.65	0.34	26941265	63478415	0.51
4	3	51.77	0.35	13744055	35960665	0.26

Lane	Band No.	Mol. Wt. (KDa)	Relative Front	Adj. Volume (Int)	Volume (Int)	Rel. Quant.
4	4	50.07	0.37	4349930	28886585	0.08
4	5	47.88	0.39	1859455	19774430	0.03
4	6	46.11	0.41	17698265	50316045	0.33
4	7	45.39	0.43	19614205	42177070	0.37
4	8	44.99	0.44	1350635	12687155	0.03
4	9	44.42	0.46	3670225	25050870	0.07
5	1	62.29	0.26	1714960	12419440	0.03
5	2	54.00	0.33	62441120	95498480	1.17
5	3	52.00	0.35	30574880	41040800	0.57
5	4	49.85	0.37	29331680	39884000	0.55
5	5	48.31	0.39	26460400	34812640	0.50
5	6	46.73	0.40	56132720	71462320	1.05
5	7	45.48	0.42	42652960	55819440	0.80
5	8	45.11	0.44	12349040	20932000	0.23
5	9	44.57	0.46	17721520	35045680	0.33
6	1	62.01	0.26	2771496	14996196	0.05
6	2	53.76	0.33	76422376	96913812	1.43
6	3	52.35	0.35	48208276	61203118	0.90
6	4	50.18	0.37	40501686	51889034	0.76
6	5	48.64	0.38	36684440	46411748	0.69
6	6	47.15	0.40	66937682	83946512	1.26
6	7	45.58	0.42	53287378	68713154	1.00
6	8	45.17	0.43	19302148	30302592	0.36
6	9	44.63	0.45	25955092	48248602	0.49
7	1	45.55	0.42	1280760	11552190	0.02
7	2	44.62	0.45	5850078	22613136	0.11
9	1	54.37	0.33	1446432	10855416	0.03
10	1	55.10	0.32	1615614	12870468	0.03
10	2	45.58	0.42	1639404	11370528	0.03
11	1	54.86	0.32	22450350	48325446	0.42
11	2	52.46	0.35	10978110	30462120	0.21
11	3	49.30	0.38	650130	9553362	0.01
11	4	47.36	0.40	3257670	20090616	0.06
11	5	45.66	0.42	13784004	33567378	0.26
11	6	44.75	0.45	1991418	15008058	0.04

Figure S3. Standard Curve BSA of Quantification of Purified Proteins

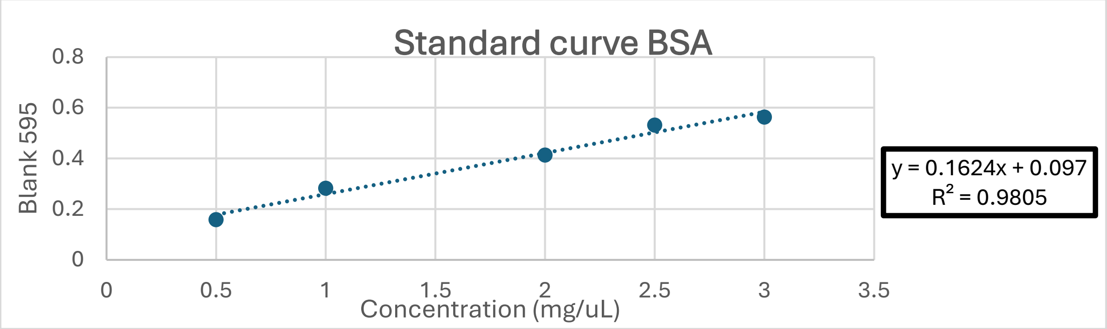

Table S6. Quantification of Purified Proteins Concentration by the Bradford Method

Prot_689			Prot_504		
#	Concentration (mg/µl)	Fractions	#	Concentration (mg/µl)	Fractions
0	1.50	PT	0	2.08	PT
1	0.46	PI1	1	1.99	PI1
2	0.36	PI2	2	0.88	PI2
3	0.02	W3	3	0.00	W3
4	0.00	W4	4	0.00	W4
5	0.08	E7	5	0.00	W7
6	0.14	E8	6	0.23	WE8
7	0.36	E9	7*	0.64	E9
8*	0.87	E10	8	0.49	E10
9	0.00	EW11	9	0.08	EW11
10	0.00	EW12	10	0.00	EW12
11	0.00	EW13	11	0.00	W13
12	0.00	EW14	12	0.00	W14
13	0.00	W15	13	0.00	W15
14	0.00	W16	14	0.00	W16
15	0.00	W17	15	0.00	W17

*Fraction with the highest concentration of purified recombinant protein, corresponding to the eluate.

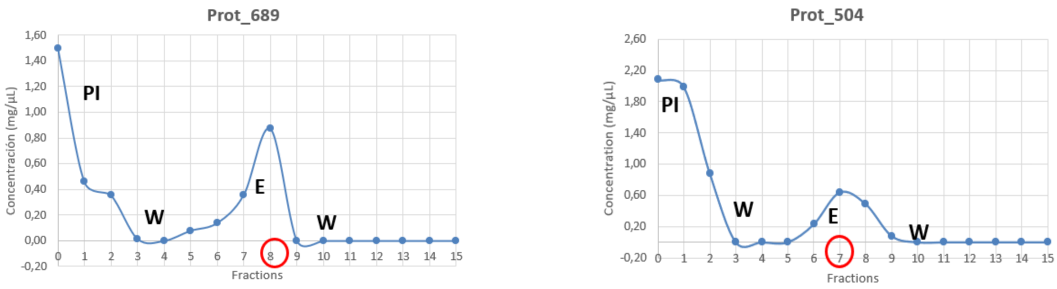

Figure S4. Purification Fractions Collected and Concentration Profiles. PI: Non-specific Protein. W: Wash Fraction. E: Eluted Fraction. The fraction with the highest concentration is highlighted by a red circle.

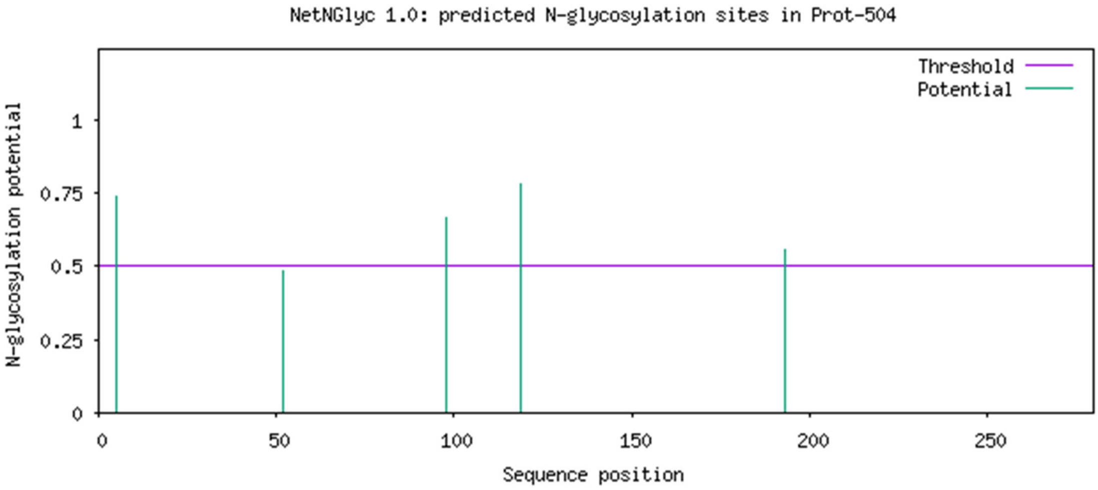

*Signal Peptide from Amino Acid 1-38

Figure S5. Predicted N-glycosylation Sites in Prot_504 by NetNGlyc 1.0.
